# miRNAs Related to Different Processes of Fracture Healing: An Integrative Overview

**DOI:** 10.3389/fsurg.2021.786564

**Published:** 2021-11-19

**Authors:** Rald V. M. Groven, Johan van Koll, Martijn Poeze, Taco J. Blokhuis, Martijn van Griensven

**Affiliations:** ^1^Department of Cell Biology-Inspired Tissue Engineering, MERLN Institute for Technology-Inspired Regenerative Medicine, Maastricht University, Maastricht, Netherlands; ^2^Division of Traumasurgery, Department of Surgery, Maastricht University Medical Center, Maastricht, Netherlands

**Keywords:** miRNAs, fracture healing, bone regeneration, osteogenic differentiation, angiogenesis, hypoxia

## Abstract

Fracture healing is a complex, dynamic process that is directed by cellular communication and requires multiple cell types, such as osteoblasts, osteoclasts, and immune cells. Physiological fracture healing can be divided into several phases that consist of different processes, such as angiogenesis, osteogenesis, and bone resorption/remodelling. This is needed to guarantee proper bone regeneration after fracture. Communication and molecular regulation between different cell types and within cells is therefore key in successfully orchestrating these processes to ensure adequate bone healing. Among others, microRNAs (miRNAs) play an important role in cellular communication. microRNAs are small, non-coding RNA molecules of ~22 nucleotides long that can greatly influence gene expression by post-transcriptional regulation. Over the course of the past decade, more insights have been gained in the field of miRNAs and their role in cellular signalling in both inter- and intracellular pathways. The interplay between miRNAs and their mRNA targets, and the effect thereof on different processes and aspects within fracture healing, have shown to be interesting research topics with possible future diagnostic and therapeutic potential. Considering bone regeneration, research moreover focusses on specific microRNAs and their involvement in individual pathways. However, it is required to combine these data to gain more understanding on the effects of miRNAs in the dynamic process of fracture healing, and to enhance their translational application in research, as well as in the clinic. Therefore, this review aims to provide an integrative overview on miRNAs in fracture healing, related to several key aspects in the fracture healing cascade. A special focus will be put on hypoxia, angiogenesis, bone resorption, osteoclastogenesis, mineralization, osteogenesis, osteoblastogenesis, osteocytogenesis, and chondrogenesis.

## Introduction

Fracture healing is a complex, dynamic process that is directed by cellular communication and requires multiple cell types, such as osteoblasts, osteoclasts, and immune cells. Physiological fracture healing can be divided into several phases that consist of different processes, such as angiogenesis, osteogenesis, and bone resorption/remodelling, in order to guarantee proper bone regeneration after fracture (1). Cellular communication and molecular regulation are important in orchestrating these processes to enable swift interplay and transitioning among them. Disturbance of the multiphase fracture healing cascade can result in healing impairments such as delayed unions or non-unions. Such healing impairments often require treatment through multiple surgical interventions followed by long rehabilitation periods and, in case treatment does not suffice, can have invalidating consequences for the patient (2). Therefore, understanding the cellular communication and molecular regulation in and around the fracture zone is of great importance.

MicroRNAs (miRNAs) are small, non-coding, double stranded RNA molecules that function as important post-transcriptional regulators in both inter- and intracellular signalling. MiRNAs were discovered as early as 1993 by Lee et al. and have been under increasing attention in the health and life sciences thereafter (3). Let-7, one of the miRNAs that is discussed in the present review, was one of the first that was shown to be involved in the development of larval stages in *C. elegans* (4, 5). Their primary mechanisms of action are cleavage of protein translations or direct repression of target messenger RNAs (mRNAs). Pre-miRNAs are generated in the nucleus after which they are transported into the cytoplasm through exportin-5. Pre-miRNAs are then cleaved into mature miRNAs by dicer, after which they bind to the RNA-induced silencing complex (RISC) which acts as the functional unit to repress target mRNAs. Imperfect complementary binding between the miRNA and its target mRNA leads to translational inhibition whereas near perfect complementary binding results in cleavage of the target mRNA (6). To date, 2,656 mature, human miRNAs have been identified (miRbase version 22; http://mirbase.org/).

MiRNAs have been broadly researched in endocrinology, cardiology, and oncology, and are under increasing attention in the field of bone regeneration over the past decade (7–9). For example, patient-based work by Seeliger et al. and Kelch et al. has shown that miRNAs are linked to osteoporosis and osteoporotic fractures in both serum as well as bone samples (10, 11). In fact, miRNAs are involved in various musculoskeletal pathophysiological mechanisms (12). Looking at physiological fracture healing, recent *in vitro* studies have shown that miRNAs can influence processes that are involved in bone regeneration, such as angiogenesis and osteogenesis (13, 14). Post transcriptional regulation by miRNAs can be complex, since one single miRNA can directly target several mRNAs. Integrating the effects of miRNAs on the different processes taking place within the fracture healing cascade can therefore be challenging.

The potential clinical application of miRNAs in the field of bone regeneration is greatly dependent on the understanding of miRNA expression and target analysis. As illustrated above, fracture healing consists of various processes in which miRNAs play a role. Even though much research has been done on miRNAs in specific processes within fracture healing, an overview that summarises up to date data on miRNAs in these specific processes is lacking in current literature.

Therefore, this review aims to provide an integrative overview on miRNAs in fracture healing, related to several key aspects in the fracture healing cascade. A special focus will be put on hypoxia, angiogenesis, bone resorption, osteoclastogenesis, mineralization, osteogenesis, osteoblastogenesis, osteocytogenesis, and chondrogenesis.

## Literature Search and Selection

A systematic search of the database PubMed was performed to identify relevant articles. Search keywords were (osteogenesis OR osteogenic) AND (regulation OR regulatory) AND (mirna OR microrna). To be included in this review, papers had to be listed in PubMed, written in the English language and present data of original research. Two independent researchers (RVMG and JvK) performed the selection of included literature. A first selection was made by title and abstract. Articles were then grouped into sections regarding the different fracture healing aspects as described above. Reviews and articles that dealt with long non-coding RNAs, circular miRNAs, pathological fracture healing, glucocorticoid induced osteogenic differentiation, deregulated inflammation, fracture healing impairments, osteonecrosis, osteoporosis, periodontal Bone Mesenchymal Stem Cells (BMSCs), adipogenic Mesenchymal Stem Cells (MSCs), or vascular calcification were not included in this review.

## Results

All miRNAs that are discussed throughout this review are summarised in Table 1 according to the aspects of fracture healing and their involvement therein.

**Table 1:** miRNAs and their involvement in different aspects of fracture healing.

**microRNAs**	**Hypoxia**	**Angiogenesis**	**Bone resorption**	**Osteoclastogenesis**	**Mineralization**	**Osteogenesis and** **osteoblastogenesis**	**Osteocytogenesis**	**Chondrogenesis**
miR-21	↑		↑	↑ ^*^	↑	↑	↑	
miR-128				↑	↑	**^~^**		
miR-23a				↑	↑ ^*^	^~^ ^*^	↑	
miR-27a					↑ ^*^	^~^ ^*^	↑	
miR-143		↑ ^*^				^~^ ^*^		
miR-218				↓	↑	↑		↑
miR-222		↓ ^*^				↑ ^*^		^~^ ^*^
miR-29a				↓	↑ ^*^	↑ ^*^		↑ ^*^
miR-223					↑ ^*^	^~^ ^*^		
miR-126		↑ ^*^			↑			
miR-135b					↑ ^*^	↑ ^*^		
miR-140						↑ ^*^		↑
miR-142				^~^ ^*^	↑	↑		
miR-23b				↑		↑		
miR-26b						**^~^**		↓
miR-31a			↑	↑				
miR-335						↑		↑
miR-451					↑	↑		
miR-503				↓	↑	↑		
miR-9				↓	↑	↑		
miR-296					↑ ^*^	↑ ^*^		
miR-34c				↑	↑	↓		
let-7						↑ ^*^		
let-7f						↑ ^*^		
miR-1	↑							↓ ^*^
miR-125a				↑		↓ ^*^		
miR-135	↑							
miR-146a						↑ ^*^		↓ ^*^
miR-150					↑	↓ ^*^		
miR-155	↑ ^*^				↓	↓		
miR-15b						↑ ^*^		
miR-181a						↓ ^*^		↑ ^*^
miR-182				↑		↓		
miR-183				↑				
miR-199a	↑ ^*^							
miR-24							↑	
miR-25						↑		
miR-26a		↑ ^*^						
miR-337								↑
miR-409					↓	↑		
miR-429	↑							
miR-483				↑ ^*^				
miR-675	↑ ^*^							
miR-98						↑ ^*^		
miR-99				↑ ^*^				
miR-346						↑ ^*^		
miR-664						↑ ^*^		
miR-149						↑		
miR-187						↑ ^*^		
miR-194						↑		
miR-219						↑		
miR-224						↑ ^*^		
miR-302a						↑		
miR-877						↑		
miR-5106						↑		
miR-100			↓	↓				
miR-10a		↓						
miR-124				↓	↓	↓ ^*^		
miR-125b					↓ ^*^	↓ ^*^		↓ ^*^
miR-137		↓						
miR-145				↓		↓		
miR-148a					↓			
miR-181				↓				
miR-191		↓						
miR-195		↓ ^*^						
miR-200b		↓ ^*^						
miR-214						↓ ^*^		↓ ^*^
miR-29b					↓ ^*^			
miR-30c2					↓ ^*^			
miR-338				↓				
miR-92a		↓						
miR-206						↓ ^*^		↓
miR-10					↓	↓		
miR-10b						↓		
miR-17					↓	↓		
miR-22						↓		
miR-23						↓ ^*^		
miR-31						↓ ^*^		
miR-103					↓ ^*^	↓ ^*^		
miR-383						↓		
miR-205					↓	↓		
miR-449b						↓		
miR-34a						↓ ^*^		
miR-203						↓ ^*^		
miR-320b						↓ ^*^		
miR-153						↓ ^*^		
miR-204						↓ ^*^		
miR-211						↓		
miR-765						↓ ^*^		
miR-342						↓ ^*^		
miR-542					↓	↓		
miR-133a					↓	↓ ^*^		
miR-217						↓		
miR-375						↓		
miR-505						↓		
miR-138					↓ ^*^	↓ ^*^		
miR-139					↓ ^*^	↓ ^*^		
miR-545						↓		
miR-193a					↓ ^*^	↓ ^*^		
miR-141					↓ ^*^	↓ ^*^		
miR-200a					↓	↓		
miR-144						↓		
miR-186					↓	↓		
miR-188						↓		
miR-363						↓		
miR-384						↓		
miR-532					↓	↓		
miR-320a					↓ ^*^	↓ ^*^		

*Green upward arrows depict a positive effect of the specific miRNA on the aspect related to fracture healing. Red downward arrows depict a negative effect of the specific miRNA on the aspect related to fracture healing.*

~ *depicts controversial effects regarding the specific miRNA on the aspect related to fracture healing.*

* *depicts that the specific miRNA in the relevant aspect has been researched in a human setting*.

### Hypoxia

Disturbed vascularization causes hypoxia in the fracture zone during the initial phase of the fracture healing cascade (2). Hypoxia can induce gene expression through a set of transcription factors that respond to low oxygen levels, the so-called Hypoxia Inducible Factors (HIFs) (15). Adequate blood supply is key in proper fracture healing and is therefore important to restore normoxic conditions as soon as possible after bone fracture to stimulate bone regeneration. In fact, poor blood supply and prolonged hypoxia are risk factors for the development of fracture healing impairments (15). HIFs play an important role in many cellular processes of interest. Several miRNAs have been specifically researched in the light of hypoxia and fracture healing. MiR-1, miR-21, miR-135, miR-155, miR-199a, miR-429, and miR-675 have all been shown to enhance bone regeneration through pathways that are regulated via hypoxia.

MiR-1 and miR-135 enhanced bone regeneration by directly targeting Hypoxia Inducible Factor 1 Subunit Alpha Inhibitor (HIF1AN), resulting in enhanced expression of osteogenic markers such as Runt-related transcription factor 2 (RUNX2), Alkaline Phosphatase (ALP), Osteocalcin (OCN), Osterix (OSX), and Osteopontin (OPN) (16, 17). MiR-21 downregulated the expression of Small Mothers Against Decapentaplegic (SMAD) 7 in hypoxic conditions. Under normal conditions, SMAD7 suppressed the expression of RUNX2. Therefore, miR-21 promoted bone regeneration through enhancing RUNX2 expression (18). In hypoxic conditions, miR-155 and miR-199a negatively regulated Hypoxia Inducible Factor 1 Subunit Alpha (HIF-1A) and showed to enhance bone regeneration both *in vitro*, as well as *in vivo* (19, 20). MiR-429 enhanced osteoblast differentiation both *in vitro* as well as *in vivo*. Apart from enhancing osteoblastic differentiation *in vitro*, miR-429 also accelerated bone formation and remodelling *in vivo* (21).

Lastly, miR-675 showed to enhance osteoblastic differentiation through increasing HIF-1A response, whilst simultaneously activating the Wingless-related integration site signalling (Wnt-signalling) pathway. Depletion of miR-675 in human MSCs (hMSCs) under hypoxic conditions decreased the differentiation capacity of hMSCs toward the osteoblastic lineage, and reduced angiogenesis as described below (22).

### Angiogenesis

Adequate blood supply to the fracture zone is necessary to ensure a proper supply of nutrients, cells, and signalling molecules, but also to normalise the acidic and hypoxic conditions that occur after trauma due to reduced vascularization (1). Various stimuli can trigger angiogenesis, such as hypoxia, tissue damage, nutrient demand or cellular growth, and proliferation. In the field of bone regeneration, miR-26a, miR-126, and miR-143 have shown to exert pro-angiogenic effects.

Although the exact mechanism of action has not yet been elucidated, miR-26a enhanced angiogenesis by increasing the expression of both Vascular Endothelial Growth Factor (VEGF) and Angiopoietin 1 (Ang1). Besides pro-angiogenic effects, miR-26a also showed to elicit pro-osteogenic effects through upregulating RUNX2, OCN, Bone Morphogenic Protein 2 (BMP2), and Collagen type 1 Alpha 1 (COL1A1) (23). *In vivo* research has shown that miR-126 increased the expression of angiogenesis related genes in fracture healing, such as HIF-1A, Ang1, VEGF-A, and Transforming Growth Factor Beta 1 (TGF-β1) (24). MiR-143, also involved in osteogenesis, promoted angiogenesis via the Wnt-signalling pathway through directly targeting Histone Deacetylase (HDAC) 7, which inhibits endothelial growth through binding with β-catenin. This has been shown *in vivo* where administration of miR-143 promoted angiogenesis, as well as osteogenesis (25). Contrarily, miR-10a, miR-92a, miR-191, miR-195, miR-200b, and miR-222 inhibited angiogenesis. MiR-10a exerted its anti-angiogenic effects by inhibiting VEGF and β-catenin expression, thereby interfering with angiogenesis through these two important mediators (26). MiR-92a has anti-angiogenic capacities by targeting Integrin Alpha 5 and Mitogen activated protein Kinase Kinase 4. Work by Murata et al. revealed that miR-92a was downregulated during normal fracture healing. It was hypothesised that further downregulation of miR-92a might aid in enhancing angiogenesis (27). The capability of MSCs to migrate and invade tissue is important in angiogenesis. MiR-191 interferes with these capabilities, as it targets both Matrix Metallo Proteinase (MMP) 9 and MMP13. Furthermore, miR-191 reduced VEGF expression, similar to miR-195 (28, 29). MiR-200b has been shown to downregulate the VEGF receptor Kinase Insert Domain Receptor, as well as reducing the expression of v-Ets Erythroblastosis Virus E26 Oncogene Homologue 1 (ETS1), GATA binding protein 2 and Zinc Finger E-Box Binding Homeobox 2, involved in VEGF-mediated angiogenesis (30). *In vivo* work by Yoshizuka et al. showed that miR-222 reduced capillary density in a murine fracture healing model. Although the exact mechanism has not yet been validated, pathway analysis identified the tyrosine-protein kinase receptor, together with Signal Transducer and Activator of Transcription 5A (STAT5A), as direct targets of miR-222 (31). Lastly, miR-137 might inhibit angiogenesis as silencing miR-137 enhanced angiogenesis through directly targeting Stromal Cell-Derived Factor 1a (SDF-1a), a well-known promotor of angiogenesis (32).

### Bone Resorption

Bone is constantly resorbed and renewed, both after bone fracture, as well as in normal bone remodelling (1). Therefore, bone resorption is considered an important aspect for bone regeneration. Bone resorption is primarily dependent on both the number of osteoclasts present in the bone, as well as their functional activity. The number of osteoclasts is dependent on osteoclastogenesis, whereas their functional activity depends on various parameters, such as mechanical strain on the bone, as well as patient-related factors such as nutritional state and lifestyle. In this section, the latest literature in relation to the involvement of specific miRNAs in bone resorption is discussed.

Osteoclast functional activity was enhanced by miR-21 and miR-31a. Hu et al. examined the effect of miR-21 on osteoclast functioning and osteoclastogenesis in mice. They showed that miR-21 targeted Programmed Cell Death Protein 4, resulting in enhanced bone resorption, as well as enhanced osteoclastogenesis (33). MiR-31a showed to promote bone resorption and osteoclastogenesis *in vivo* by targeting Ras Homologue family member A (RhoA). Impairments in bone resorption were observed upon increased RhoA expression. Therefore, miR-31a is deemed to enhance osteoclast functioning by targeting RhoA (34).

Contrarily, *in vivo* work by Zhou et al. showed that miR-100 suppressed bone resorption, as well as osteoclastogenesis. Although the exact mechanism has not yet been confirmed, this effect is most likely due to targeting Fibroblast Growth Factor 21 by miR-100. Furthermore, miR-100 overexpression has shown to effectively block OVX-induced bone resorption, emphasising its role in bone resorption as well as its therapeutic potential in osteoporosis (35). Kelch et al. examined miRNAs in bone tissue, osteoblasts, and osteoclasts from osteoporotic patients. Surprisingly, they found that miR-100 was significantly upregulated in isolated osteoblasts and osteoclasts from osteoporotic patients, indicating that miR-100 might be expressed to a greater extent to protect against failing bone homeostasis (11).

### Osteoclastogenesis

Fracture healing is a dynamic process which consists of both the production of new bone in order to bridge the fracture gap, as well as the resorption and remodelling of bone. In order to maximally regain the original morphology and proper function of a bone, remodelling is required and takes place over a period of time that can extend long after the initial phases of fracture healing. Osteoclasts originate from monocytes, and are the major cell type that facilitate this resorption and remodelling of bone (1). Their formation and development, osteoclastogenesis, is also under the influence of miRNAs. Specifically, miR-21, miR-31a, miR-34c, miR-99, miR-125a, miR-128, miR-142, miR-182, miR-183, and miR-483 have shown to enhance osteoclastogenesis.

MiR-21, a miRNA that has proven to be involved in various aspects of bone regeneration, enhanced osteoclastogenesis. It targeted Sprouty RTK Signalling Antagonist 1 and Phosphatase and Tensin Homologue (PTEN), thereby reducing Extracellular signal-Regulated Kinases (ERK) activity whilst activating Phosphatidylinositol 3-Kinase/serine threonine kinase (PI3K/AKT) signalling, leading to enhanced osteoclastogenesis and bone resorption (33, 36). MiR-21 has also shown to prevent osteocyte apoptosis, which is known to stimulate osteoclastogenesis (37). *In vitro* work by Xu et al. revealed the pro-osteoclastogenic properties of miR-31a. Apart from enhancing osteoclastogenesis through enhanced RhoA expression, it also promoted osteoclast functioning and inhibited osteoblastogenesis (34). *In vivo* work by Bae et al. revealed an interesting interaction by which miR-34c targeted multiple components of the Notch signalling pathway, thereby enhancing osteoclastogenesis, whilst suppressing osteoblastogenesis (38).

Exhibiting similar characteristics, miR-99 promoted osteoclastogenesis whilst simultaneously inhibiting osteogenic differentiation by suppressing the expression of RUNX2 and ALP, whereas osteogenic fusion markers Dendritic Cell-Specific Transmembrane Protein, C-C motif chemokine ligand 2 and osteogenic-specific marker Cathepsin K (CTSK) were increasingly expressed (39). An interesting miRNA with regard to osteoclastic differentiation and functioning is miR-125a. It exerted a dual effect on osteoclasts by promoting osteoclastic differentiation through directly targeting Tumour Necrosis Factor (TNF) Receptor Superfamily Member 1B, whilst simultaneously enhancing osteoclast motility by increasing MMP expression (40). Activated in part by TNF, Nuclear Factor Kappa-light-chain-enhancer of activated B cells (NF-κB) signalling is also required in osteoclastogenesis. MiR-128 engages with this signalling pathway to enhance osteoclastogenesis by directly targeting Sirtuin 1 (Sirt1) which, under normal circumstances, inhibits NF-κB signalling. The positive effect of miR-128 is underlined by the increased expression of osteoclastogenic marker Nuclear Factor of Activated T-cells (NFATc) 1 both *in vitro* as well as *in vivo* (41). Work by Inoue et al. showed that miR-182 exerted pro-osteoclastogenic effect by targeting Protein Kinase R, an inhibitor of osteoclastogenesis through suppression of the Interferon-Beta pathway. Several osteoclastic markers, among others NFATc1 and Tartrate-Resistant Acid Phosphatase, showed increased expression levels *in vitro* upon increased miR-182 levels, whereas *in vivo* miR-182 knockout resulted in decreased expression levels of osteoclastic marker genes (42, 43).

MiR-183 increased osteoclastogenesis by direct targeting of Heme-oxygenase-1, which subsequently resulted in enhanced Receptor Activator of NF-κB Ligand (RANKL) signalling (44). Lastly, *in vivo* work by Li et al. revealed the regulatory role of miR-483 in osteoclastogenesis. MiR-483 reduced osteoclast apoptosis whilst enhancing the expression of osteoclastic differentiation markers, such as NFATc1, NFATc2, and CTSK (45). A miRNA of particular interest regarding osteoclastogenesis is miR-142. MiR-142 showed to exert both pro- as well as anti-osteoclastogenic effects, affecting various cellular communication processes. Osteoclast differentiation is promoted by miR-142 through directly targeting PTEN and thereby enhancing the activity of the PI3K/Akt/Forkhead box O1 (FOXO1) pathway (46). Contrarily, Fordham et al. describe a mechanism by which overexpression of miR-142 reduced osteoclastic differentiation and induced osteoclast apoptosis through RANK-signalling (47).

Osteoclastogenesis was inhibited by miR-9, miR-29a, miR-100, miR-124, miR-142, miR-145, miR-181, miR-218, miR-338, and miR-503. Both miR-9, as well as miR-181 have shown to directly target Casitas B-lineage Lymphoma and lead to reduced ubiquitination of B-Cell Lymphoma 2 (Bcl2) like protein 11, an important pro-apoptotic protein, through which both miRNAs enhanced osteoclast apoptosis (48). Osteoclastogenesis is under regulation of RANKL and CXC motif chemokine 12, both being repressed by miR-29a. Interestingly, miR-29a simultaneously enhanced osteoblast functioning, as discussed in section Osteogenesis and Osteoblastogenesis (49). Apart from regulating bone resorption, as described in section Bone Resorption, miR-100 inhibited osteoclastogenesis *in vivo* by reducing the expression levels of Fibroblast Growth Factor 21 (35). MiR-124 inhibited osteoclast formation via direct targeting of NFATC1, reducing the expression of NFATC1 dependent genes, such as Acp5 and CTSK. Apart from inhibiting osteoclastogenesis, miR-124 also reduced osteoclast motility through reduced expression of RhoA and Rac Family Small GTPase1 (Rac1) (50, 51).

One of the major signal transducers in osteoclastogenesis, SMAD3, showed to be directly targeted by miR-145, thereby inhibiting the downstream expression of osteoclastic genes, such as NFATC1, Tartrate-Resistant Acid Phosphatase and CTSK (52). As described previously in this section, the NF-κB pathway is an important signalling pathway in osteoclast formation, which is activated by TNF. MiR-218 interfered with this signalling pathway through direct targeting of the TNF-membrane receptor, thereby inhibiting the activation of the NF-κB pathway and reducing osteoclastogenesis (53). Through a partially similar mechanism, miR-338 affected the formation and activity of osteoclasts by targeting I-Kappa B Kinase, a master regulatory kinase that activated the NF-κB signalling pathway (54). Lastly, through directly targeting RANK, miR-503 inhibits RANK-mediated osteoclastogenesis (55).

### Mineralization

Bone can be divided into two main components, an organic- and an inorganic component. The inorganic component of bone, which constitutes roughly 60% of the total bone mass, mainly consists of hydroxyapatite. The organic component of bone, the remaining 40%, consists of proteins, such as collagens, and water (56). Collagens warrant a certain degree of flexibility in the bone whereas the hydroxyapatite mainly aids in withstanding axial stress. Mineralization of the collagen rich network is therefore an important aspect in bone regeneration. Even though many miRNAs influence collagen expression, several miRNAs have been researched with a special emphasis on matrix mineralization. MiR-21, miR-126, miR-29a, miR-142, miR-150, miR-218, and miR-451 have shown to enhance matrix mineralization. Increased miR-21 expression has shown to enhance matrix mineralization in several *in vitro* and *in vivo* studies. It does this by targeting SMAD7, thereby enhancing matrix mineralisation through increased expression of RUNX2, ALP, OCN, and OPN (57–59). MiR-21 also enhanced the expression of Myocyte Enhancer Factor 2c, a protein that under normal conditions inhibits Sclerostin (SOST). On its turn, SOST affects osteoblast-mediated bone formation through matrix mineralization and inhibits cellular differentiation toward the osteogenic lineage (60). In short, miR-21 has several points of engagement in matrix mineralization, as well as other aspects of bone regeneration. Similar to miR-21, miR-126 enhanced matrix mineralization by targeting SOST (61, 62).

MiR-29a showed to enhance matrix mineralization *in vitro* by targeting Dickkopf-related protein 1 (DKK1), kremen and Secreted Frizzled-Related Protein 2 (SFRP2), thereby upregulating Wnt-signalling. This promoted matrix mineralization and increased expression of osteogenic markers, such as BMP2, OPN, OCN, and COL1A1 (63).

MiR-142, important in cellular differentiation toward both the osteoblastic- as well as the osteoclastic lineage, has shown to enhance mineralization by targeting WW domain containing E3 ubiquitin protein ligase 1, a negative regulator of osteoblast differentiation. MiR-142 downregulation reduced the expression of RUNX2, OCN, and JunB, a well-known cell proliferation inhibitor (64).

Mineralization was also enhanced by increased expression of miR-150. MMP14, a key enzyme in cellular migration, invasion, proliferation, and bone resorption, is a direct target of miR-150 and therefore potentially enhances osteoblast functioning (65). Proven to be involved in chondrogenesis as described in section Osteogenesis and Osteoblastogenesis, miR-218 also increased mineralization nodule formation (66). MiR-451, a glucose regulated miRNA, enhanced mineralization *in vitro* and *in vivo* through suppressing Odd Skipped Related 1 whilst enhancing RUNX2 and BMP-4 expression (67).

MiR-29b, miR-30c2, and miR-125b inhibited mineralization both *in vitro* as well as *in vivo*. Research has shown that various osteogenic markers are upregulated in response of their inhibition (68, 69). Apart from its involvement in osteoclastogenesis, as described in section Osteocytogenesis, *in vitro* work by Moura et al. showed that miR-99 reduced matrix mineralization (39). Similarly, miR-148a decreased ALP activity, osteogenic marker expression and subsequently reduced matrix mineralization (70). MiR-409 inhibited matrix mineralization through targeting Low-density Lipoprotein Receptor-Related Protein (LRP) 8 and decreasing ALP activity. Conversely, inhibiting miR-409 promoted osteoblast functioning (71).

### Osteogenesis and Osteoblastogenesis

Osteoblasts are among the most important cells in bone regeneration. They synthesise bone matrix and coordinate mineralization of the skeleton. Together with osteoclasts and osteocytes, both discussed in more detail in later sections of this review, they regulate bone homeostasis and regeneration (1, 72). In this section, the role of miRNAs within osteogenesis and osteogenic differentiation is discussed, putting special focus on osteoblastogenesis.

Involved in various cell types, the Wnt-signalling pathway also regulates osteogenic differentiation and is under the influence of miRNAs. Under normal circumstances, β-catenin is continuously degraded by the axin complex. Let-7f has shown to directly target axin 2 in hMSCs, thereby enhancing osteogenic differentiation through promoting β-catenin activity (73). Let-7, a miRNA closely related to let-7f, has shown to promote osteogenic differentiation by directly targeting Chemokine-Like Receptor 1. Chemokine-Like Receptor 1 normally mobilises intracellular calcium and activates signalling cascades, such as NF-κB (74). Although the exact mechanism is not yet fully known, osteoblastogenesis and osteoblast functioning were enhanced by miR-9, as shown by *in vitro* work from Luo et al. where the expression levels of RUNX2 and mineralization were enhanced upon miR-9 overexpression (75). MiR-15b indirectly enhanced osteogenic differentiation by preventing the degradation of RUNX2, through directly targeting SMAD Ubiquitylation Regulatory Factor (SMURF) 1 (76). MiR-21 is also involved in osteogenesis and is of particular interest, since it is involved in various aspects of bone regeneration as described throughout this review. MiR-21 targets SMAD7, a well-known inhibitory protein that antagonises the phosphorylation of other SMAD proteins. By doing so, miR-21 has shown to enhance osteogenic differentiation and mineralization both *in vitro* as well as *in vivo* (58).

Controversy exists regarding the role of miR-23a in osteogenesis. *In vitro* work by Guo et al. showed that miR-23a and miR-23b promoted osteogenic differentiation and mineralization, as seen by enhanced expression levels of RUNX2, OCN and ALP (77). Contrarily, Li et al. observed that miR-23a overexpression resulted in reduced osteogenic differentiation, ALP activity, and decreased expression levels of RUNX2, OPN, and Bone Sialoprotein (78). This finding is reinforced through similar observations by work from Ren et al. that report on reduced osteogenic differentiation and mineralization in hMSCs upon miR-23a overexpression (79). Presumably by enhancing Rac1 expression, miR-25 activated PI3K/AKT and c-Jun N-terminal kinase pathways, leading to enhanced osteoblastic viability and migration, as well as increased expression levels of osteogenic marker genes RUNX2 and OCN (80). Research by Jiang et al. showed that miR-25 also targeted the expression SMURF1, thereby enhancing RUNX2 signalling and subsequently promoting osteogenic differentiation (81). Hu et al. examined the interaction of miR-26b with the Wnt-signalling pathway in rat BMSCs. MiR-26b directly targeted Glycogen Synthase Kinase 3 Beta, thereby preventing the degradation of β-catenin and enhancing its signalling capabilities (82). Contrarily to this observation, Lin et al. report on decreased osteogenic differentiation upon miR-26b overexpression through targeting Oestrogen Receptor Alpha, thereby most likely reducing Wnt-signalling effectiveness (83).

MiR-346 showed to enhance osteogenesis in human (h) BMSCs in the same manner as miR-26b, by directly targeting Glycogen Synthase Kinase 3 Beta (84). MiR-142, also involved in osteoclastogenesis and matrix mineralization, showed to directly target WW domain containing E3 ubiquitin protein ligase 1, a negative regulator of osteoblast differentiation, thereby enhancing osteoblastogenesis (64).

MiR-27a has also shown to exhibit dual effects on osteogenesis. Work by Fu et al. showed that miR-27a was downregulated in serum of patients suffering from osteoporosis. Subsequent *in vitro* work on hMSCs revealed pro-osteogenic capacities of miR-27a by direct targeting of Activating Transcription Factor 3. This resulted in enhanced osteoblastic differentiation and matrix mineralization (85). On the other hand, miR-27a has shown to directly target OSX and Specificity Protein 7 (Sp7), thereby inhibiting osteogenic differentiation (86, 87).

HDAC4, an enzyme involved in several aspects of bone regeneration, is important in suppressing chondrocytes and the differentiation of osteoblasts. Tan et al. revealed that HDAC4 is targeted by miR-29a, which led to increased expression levels of RUNX2 and OCN, as well as enhanced mineralization (88). Work by Gao et al. revealed an interesting interaction between miR-98 and Mammalian High-Mobility-Group Protein AT-hook 2 (HMGA2) *in vitro*. HMGA2 can prevent differentiation of stem cells, and therefore also inhibit osteogenic differentiation. MiR-98 directly targeted HMGA2, thereby decreasing its activity and subsequently enhancing osteogenic differentiation (89). *In vitro* work by Zhang et al. showed that miR-664a promoted osteogenic differentiation of hBMSCs in a similar way as miR-98, by directly targeting HMGA2 (90).

*In vivo* work by Wang et al. revealed that overexpression of miR-128 promoted osteogenic differentiation and mineralization by increasing expression levels of ALP, RUNX2, BMP-2, and COL1A1. MiR-128 directly targeted DKK2, a Wnt-signalling pathway antagonist, and thereby enhanced Wnt-signalling (91). Another important signalling pathway in bone homeostasis is the Hippo pathway. It regulates the equilibrium between osteoclastic bone resorption and osteoblastic bone formation. MiR-135b enhances Hippo-signalling by targeting Large Tumour Suppressor Kinase and Mps One Binder protein 1B, both important negative regulators of the Hippo-signalling pathway. By doing so, it promoted both osteogenic differentiation and mineralization, as seen by increased expression levels of ALP, OSX, RUNX2, and OPN (92).

The attraction of MSCs is also an important aspect in bone regeneration. This is in part regulated by TGF-β, which enhances both the recruitment, as well as proliferation of MSCs during bone formation. Surprisingly, TGF-β inhibits osteoblast differentiation, which emphasises the presumably delicate, time related activity of TGF-β in order to attract MSCs without preventing them from differentiating into functional osteoblasts. MiR-140 directly targets TGF-β3, thereby theoretically enhancing osteoblast differentiation. Interestingly, miR-140 overexpression resulted in increased expression levels of OCN, but did not influence the expression of ALP, COL1A1, or RUNX2 (93). As described later in this review, chondrogenesis is an important aspect of bone regeneration. MiR-146a has shown to affect chondrogenesis, whilst enhancing osteogenesis through downregulation of SMAD2 and SMAD3 protein expression, resulting in reduced SRY-Box transcription factor 9 (SOX9) expression whilst enhancing RUNX2 expression (94). MiR-149 has shown to regulate the switch between adipogenic or osteogenic differentiation of BMSCs. It presumably does this by targeting the Fat Mass and Obesity-associated protein, showing enhanced osteogenic differentiation upon miR-149 overexpression (95). Primarily researched in the field of oncology, miR-187 has also shown to enhance osteoblastic differentiation by targeting Intercellular Adhesion Molecule 1, leading to enhanced expression levels of RUNX2, OSX, and OPN (96). An important regulator of MSC lineage differentiation is Chicken Ovalbumin Upstream Promoter-transcription factor 2 (COUP-TFII). Under normal circumstances, COUP-TFII promotes the expression of Peroxisome Proliferator-Activated Receptor gamma (PPARy) whilst inhibiting RUNX2 activity and thus stimulating adipogenic differentiation. Work by Jeong et al. showed that COUP-TFII is presumably targeted by miR-194, thereby promoting osteogenic differentiation through enhanced activity of RUNX2 (97). MiR-218, involved in chondrogenesis, matrix mineralization, and osteoclastogenesis, directly targeted Hydroxyprostaglandin Dehydrogenase (HPGD) through which it enhanced the expression of several osteogenic marker genes, such as RUNX2, ALP, COL1A1, OCN, and OPN (98).

*In vitro* work by Aquino-Martinez et al. revealed a direct interaction between miR-219a and RAR-related Orphan Receptor beta. MiR-219a ostensibly targets RAR-related Orphan Receptor beta in a direct manner, thereby enhancing osteoblastic differentiation (99). Although an exact mechanism of action has to be clarified, Yoshizuka et al. showed that miR-222 enhanced bone formation and chondrogenesis, whilst reducing angiogenesis *in vivo* (31).

Similar to miR-29a, miR-223 enhances osteoblastic differentiation by targeting HDAC2. MiR-223 mimics increased the expression levels of ALP, OCN, and RUNX2. Additionally, miR-223 mimics enhanced matrix mineralization (100). Contrarily, *in vitro* work by Long et al. revealed FOXO3 as a target of miR-223. Although the exact mechanism has not been unravelled, expression levels of RUNX2, ALP, OCN, and SMAD4 were enhanced upon an increased expression of FOXO3. Conversely, miR-223 overexpression reduced the expression levels of the beforementioned osteogenic markers (101). MiR-143, also involved in angiogenesis, showed to directly target HDAC7, and thereby promoted osteogenic differentiation (25). Contrarily, Li et al. revealed that miR-143 directly targeted OSX, thereby suppressing osteogenic differentiation (102). MiR-224 directly targets Rac1, thereby enhancing osteogenic differentiation and expression levels of osteogenic marker genes RUNX2, OCN, OPN, and ALP (103). Interestingly, Rac1 is also targeted by miR-124, which results in reduced osteoclast motility, as described in section Bone Resorption of this review.

MiR-296 also promoted osteoblast differentiation, though no exact mechanism of action has been identified yet. Overexpression of miR-296 prevented apoptosis of osteoblasts, whilst enhancing matrix mineralization, as well as increased expression levels of osteogenic marker genes RUNX2, OCN, COL1A1, and ALP (104). Similar to miR-194, miR-302a stimulated osteoblastic differentiation by inhibiting COUP-TFII, thereby promoting RUNX2 activity. Interestingly, a positive feedback loop exists among RUNX2 and miR-302a. Under normal circumstances, RUNX2 promotes miR-302a expression, which on its turn inhibits COUP-TFII, a negative regulator of RUNX2. Therefore, increased miR-302a expression leads to reduced suppression of RUNX2 by COUP-TFII, resulting in enhanced miR-302a expression (105). Within the field of bone regeneration, various miRNAs interfere with the Wnt-signalling pathway. *In vivo* work by Zhang et al. showed that miR-335 reduced the expression of DKK1, a Wnt-signalling antagonist, thereby promoting osteogenic differentiation in hBMSCs (106). MiR-409 has shown to enhance osteogenic differentiation by activating the Wnt-signalling pathway. MiR-409 directly targeted Suppressor of Cancer Cell Invasion, thereby reducing its expression, whereas Suppressor of Cancer Cell Invasion upregulation showed to decrease osteoblastic differentiation and reduce protein expression of Wnt-signalling related genes, such as β-catenin, c-myc, and cyclin D1 (107). A glucose regulated miRNA, miR-451, enhanced osteoblastogenesis *in vitro* and *in vivo*, by suppressing Odd Skipped Related 1 whilst enhancing RUNX2 and BMP-4 expression (67).

Another important regulatory pathway in bone formation is the TGF-β/BMP signalling pathway. *In vivo* and *in vitro* work by Sun et al. showed that miR-503 promoted both osteogenic differentiation as well as mineralization by targeting SMURF1, a well-known inhibitor of TGF-β/BMP signalling (108). Like miR-503, miR-877 enhanced osteogenesis, both *in vitro* as well as *in vivo*, by promoting TGF-β signalling. It directly targets SMAD7, a negative regulator of RUNX2, bone metabolism, and the TGF-β signalling pathway (109). Lastly, miR-5106 from M2-macrophage derived exosomes has proven to enhance osteoblastic differentiation of BMSCs. This finding offers interesting perspectives since bone regeneration is in great part subject to the various immune cells that are present upon bone fracture. MiRNAs from these immune cells might therefore interfere with/aid in bone regeneration and play a role in fracture healing. Since trauma severity has already proven to affect leukocyte infiltration, fracture healing might in part be influenced by alternating leukocyte activities due to trauma severity (110, 111). Although limited research has been done on the role of Salt-Inducible Kinase (SIK) 2 and SIK3 in the field of bone regeneration, *in vitro* and *in vivo* work by Xiong et al. revealed a potential involvement of both kinases in bone regeneration. MiR-5106 overexpression, suppressing SIK2 and SIK3, showed to enhance osteogenic differentiation of BMSCs and bone regeneration in a murine fracture model (112).

In the remainder of this section, miRNAs will be discussed that negatively influence osteogenesis and osteogenic differentiation. As previously described, Luo et al. investigated the pro-osteogenic effects of miR-9 on BMSCs. In the same study, the group examined the effect of miR-10 on osteogenesis. Contrarily to miR-9, miR-10 suppressed both mineralization, as well as osteogenic differentiation capabilities by reducing β-catenin expression as well as directly targeting Bcl6 (26, 75). A family member of miR-10, miR-10b, has also proven to negatively regulate osteoblast differentiation. MiR-10b directly targets Bcl6, an important promotor of osteoblastic differentiation, whilst enhancing expression levels of STAT1, a negative regulator of osteoblastic differentiation (113). *In vitro* work by Chen et al. showed that miR-17 reduces both osteoblastic differentiation, as well as mineralization through directly targeting SOX6. Expression levels of ALP, COL1A1, and OPN were decreased upon miR-17 overexpression, whereas miR-17 inhibition led to an increased expression of these genes (114). MiR-22 has also proven to reduce the expression levels of ALP, OCN, and RUNX2, whilst enhancing apoptosis. Additionally, miR-22 showed to indirectly inactivate the p38 Mitogen-Activated Protein Kinase (MAPK)/c-Jun N-terminal kinase signalling pathway. Therefore, miR-22 suppresses both osteoblastic differentiation, as well as osteoblast viability (115). Similar to miR-22, miR-23 also inhibits osteogenic differentiation through suppressing MAPK signalling. MiR-23 targets Myocyte-Specific Enhancer Factor 2C, thereby suppressing MAPK activation and inhibiting the expression of RUNX2 (116).

*In vitro* work by Baglio et al. revealed an interaction between miR-31 and OSX, an important transcription factor that regulates the expression of several genes involved in fracture healing, such as COL1A1 and OCN. MiR-31 directly targeted OSX, leading to reduced osteogenic differentiation of BMSCs (117). The inhibitory role of miR-31 in osteogenesis is underlined by work from Deng et al. who showed that miR-31 overexpression negatively modulated Special AT-rich Sequence-Binding Protein 2 (Satb2) expression by direct targeting, leading to decreased expression levels of osteogenic marker genes RUNX2, OPN, and OCN (118). MiR-103 and miR-383 negatively regulated osteogenic differentiation by targeting Satb2, similar to miR-31. Overexpression of miR-103, as well as miR-383 decreased expression levels of ALP, RUNX2, OCN, and reduced mineralization, whilst inhibiting miR-103 and miR-383 alleviated these phenomena (119, 120). Similarly, miR-205 and miR-449b decreased expression levels of Satb2 and RUNX2, resulting in reduced osteogenic differentiation of rat (r) BMSCs (121, 122). Additionally, miR-205 inhibited the activation of ERK/MAPK signalling, further reducing osteogenic differentiation and mineralization (121).

Research has shown that cellular metabolism can also affect osteogenic differentiation. By directly targeting glutaminase, miR-206 disturbed glutamine metabolism in hBMSCs, thereby inhibiting osteogenic differentiation. Restoring glutaminase activity in miR-206 overexpressing hBMSCs reassured osteogenic differentiation (123). Glucose metabolism is also essential for MSCs to differentiate into osteoblasts, and to subsequently synthesise large amounts of extracellular matrix proteins. By directly targeting Lactate Dehydrogenase A, a key glycolysis enzyme, miR-34a suppressed osteoblast differentiation, as was depicted by reduced expression levels of ALP, OCN, and OPN (124). Apart from glucose metabolism, *in vivo* work by Chen et al. showed that miR-34a is also involved in osteogenic differentiation through directly targeting Jagged1, thereby reducing Notch-signalling (125). Like miR-34a, miR-34c inhibited osteoblast differentiation, and even showed to enhance osteoclastogenesis by targeting Notch1, thereby reducing Notch-signalling activity (38).

*In vitro* as well as *in vivo* work by Qadir et al. showed that miR-124 inhibited osteoblastic differentiation and mineralization by targeting transcription factors Distal Less Homeobox (Dlx)2, 3, and 5. Apart from osteoblastic differentiation, osteoclastic differentiation has also shown to be affected by miR-124, whilst enhancing adipogenic differentiation (126). Similar to miR-124, *in vitro* work by Laxman et al. showed that miR-203 and miR-320b directly targeted Dlx5, thereby reducing expression levels of RUNX2 and OSX (127). Besides inhibition of Dlx transcription factors, miR-124 has also shown to directly target Sp7, thereby inhibiting osteoblast differentiation (128). MiR-145 suppresses osteogenic differentiation in a similar manner by targeting Sp7, decreasing the expression levels of ALP, OCN, and COL1A1 (129). G-protein-coupled Receptor Kinase-Interacting Protein 1, a protein known to be involved in osteoblast migration and growth, is directly targeted by miR-125a. By doing so, it inhibited osteoblastic differentiation and proliferation, as shown by reduced expression levels of ALP, RUNX2, OCN, and OSX (130). MiR-125b, a family member of miR-125a, suppressed proliferative capabilities and osteogenic differentiation of hBMSCs, presumably by targeting BMP-receptor (BMPR) 1b (131, 132). MiR-125b overexpression reduced expression levels of osteogenic marker genes RUNX2, ALP, COL1A1, and OCN (131). In order for BMPR1 to become active, it needs to be phosphorylated by BMPR2. BMPR2 showed to be a direct target of miR-153, leading to reduced osteogenic differentiation capacities (133). Work by Liu et al. showed that also directly targets BMPR2, as well as RUNX2, thereby decreasing osteogenic differentiation capacities and matrix mineralization (134). Furthermore, miR-155 showed to directly target SMAD5 and inhibit several BMP-signalling pathways, such as SMAD1, Human Immunodeficiency Virus Type I Enhancer Binding Protein 2, CCAAT Enhancer Binding Protein Beta, MYO10, and RUNX2 (135). Similar to miR-155, miR-128 directly targeted SMAD5, thereby inhibiting bone formation, as shown by *in vivo* work from Xu et al. (136).

The ligands to BMPRs, BMPs, are key players in bone formation, and widely applied in scientific research for the induction of osteogenesis. *In vitro* work by Jiang et al. revealed the anti-osteogenic capacities of miR-204 by directly binding to BMP2, a BMP that is frequently used to induce osteogenesis *in vitro* and is applied in the clinic as a bone grafting material (Infuse™) (137). Apart from targeting BMP2, miR-204 directly targeted RUNX2, leading to reduced osteoblastic differentiation of BMSCs. MiR-211, a homologue of miR-204, elicited similar effects as miR-204 (138). BMP6 was a direct target of miR-765, and miR-765 overexpression reduced BMP6 expression as well as SMAD1/5/9 phosphorylation, thereby inhibiting osteogenic differentiation of hBMSCs (139). Although the exact mechanism must be clarified, miR-342 overexpression decreased expression levels of BMP7, leading to reduced viability, proliferation, and osteogenic differentiation, whilst enhancing apoptosis. Furthermore, miR-342 inhibition increased the activity of MAPK/ERK signalling (140). BMP7 was also directly targeted by miR-542, decreasing BMP7 mediated PI3K/survivin signalling, resulting in suppressed osteoblastic differentiation and mineralization (141). BMP10 showed to be directly targeted by miR-181a, thereby decreasing osteogenic differentiation, as shown by *in vitro* work from Tao et al. (142).

Work from Peng et al. and Zhang et al. independently observed that miR-133a directly targeted RUNX2 (143, 144). MiR-133a inhibited matrix mineralization, and reduced expression levels of COL1A1, OCN, OPN, and ALP (144). Like miR-133a, miR-217, miR-375, and miR-505 directly targeted RUNX2, reducing osteogenic differentiation capacities (145–147). Furthermore, overexpression of miR-217 resulted in decreased ERK/MAPK signalling (145). MiR-138 inhibited osteogenic differentiation as shown by reduced expression levels of osteogenic marker genes RUNX2, OCN, and ALP (148). *In vitro* and *in vivo* research by Eskildsen et al. showed that miR-138 inhibited osteogenic differentiation and matrix mineralization by suppressing Focal Adhesion Kinase-ERK1/2 signalling through inhibiting Focal Adhesion Kinase expression (149).

Wnt-signalling, being involved in various cell types, is key in osteogenic differentiation. MiR-139 reduces osteogenic differentiation by targeting β-catenin and Frizzled 4, leading to reduced mineralization and decreased expression levels of RUNX2, ALP, OCN, and COL1A1 (150). Like miR-139, miR-150 suppresses osteogenic differentiation by directly targeting β-catenin, as shown by *in vitro* work from Wang et al. (151). Apart from the direct inhibition of RUNX2 by miR-375, Sun et al. displayed a dual mechanism by which miR-375 influences the Wnt-signalling pathway. Both β-catenin and LRP5, an important co-receptor of the Wnt-signalling pathway, showed to be directly targeted by miR-375, thereby reducing Wnt-signalling activity (152). Interestingly, Li et al. showed that miR-545 also targeted LRP5, but not β-catenin, thereby inhibiting osteogenesis (153). By targeting both the Wnt-signalling pathway, as well as MAPK-signalling, miR-193a attenuated osteoblastic differentiation and matrix mineralization in hBMSCs. It presumably achieves this by directly targeting High Mobility Group Box 1 Protein (HMGB1), as transfection of HMGB1 in miR-193a overexpressing hBMSCs greatly restored the inhibitory effects of miR-193a on osteoblastic differentiation (154). Sangani et al. examined the role of miR-141 and miR-200a in bone regeneration. Both miRNAs exhibited anti-osteogenic characteristics, reducing osteogenic differentiation, as well as mineralization, in murine BMSCs by direct targeting of Sodium Dependent Vitamin C transporter (155). Furthermore, miR-141 has shown to directly target SDF-1, a known potentiator of osteogenic differentiation (156). SMAD proteins are also well-known for their involvement in osteogenesis. MiR-144 interferes with osteoblastic differentiation by causing cell cycle arrest of murine mesenchymal precursors at the G0/G1 phase, presumably by targeting SMAD4. SMAD4 expression was markedly downregulated upon miR-144 overexpression, inhibition of miR-144 led to opposite results (157). MiR-182 also influences osteogenic differentiation by interfering with the cell-cycle. By directly targeting FOXO1, miR-182 attenuates the capability of FOXO1 to protect the cell against Reactive Oxygen Species (ROS), thereby enhancing apoptosis and cycle arrest. *In vivo* overexpression of miR-182 has shown to impair bone formation (158).

MiR-186 has shown to decrease osteogenic differentiation, as well as mineralization, in rBMSCs through directly targeting Sirt6. Interestingly, an interaction between Sirt6 and NF-κB was observed in which Sirt6 downregulation by miR-186 resulted in enhanced phosphorylation of NF-κB. Therefore, miR-186 attenuated osteogenic differentiation by targeting Sirt6 and thereby upregulating NF-κB (159). An interesting miRNA, showing age dependent expression, is miR-188. MiR-188, increasingly expressed with progressing age, has shown to regulate the switch between osteoblastic or adipogenic differentiation from BMSCs. Overexpression of miR-188 promoted adipogenic differentiation by targeting HDAC9 and Rictor, thereby activating PPARy (160).

MiR-214 directly targeted Baculoviral IAP Repeat-Containing Protein 7, leading to enhanced expression of STAT1 and reduced osteogenic differentiation, as shown by *in vitro* work from Liu et al. (161).

*In vitro* work with hMSCs by Huang et al. showed a negative regulatory effect of miR-320a on osteogenic differentiation, as well as mineralization. It directly targeted Homeobox A10, thereby decreasing expression levels of RUNX2, ALP, and OCN (162). TNF Receptor-Associated Factor 3, an important negative regulator of the NF-κB pathway, appeared to be a direct target of miR-363, leading to reduced osteogenic differentiation, as well as increased cellular senescence (163). Involved in cellular differentiation, proliferation, and stem cell maintenance, GLI Family Zinc Finger 2 has shown to enhance osteogenic differentiation in rBMSCs. MiR-384 directly targeted GLI Family Zinc Finger 2, thereby reducing osteogenic differentiation capacities, as well as inducing cellular senescence (164). ETS are transcription factors that play a role in angiogenesis, inflammation, and bone metabolism. ETS1 potentially functions in a synergistic manner with Sp1 to promote the expression of RUNX2 (165). MiR-532 directly targeted ETS1, thereby reducing osteogenic differentiation, as well as mineralization and osteogenic marker gene expressions. Inhibiting miR-532 generated opposite results (166).

### Osteocytogenesis

In the field of bone regeneration, osteoblasts, and osteoclasts are most often researched as key functional cells in fracture repair. However, osteocytes make up 95% of bone cells in healthy, undisturbed bone. They are important, versatile cells that influence both osteoblast as well as osteoclast functioning. Osteocytes act as mechanoreceptors and thereby steer bone formation by direct cellular communication with osteoblasts and osteoclasts (167). More research is being performed on osteocytes and their role in bone regeneration. Therefore, this section focusses on miRNAs and osteocytes in relation to fracture healing.

Davis et al. researched a pathway in which osteocyte apoptosis was linked to an age dependent expression of miR-21. Although the exact mechanism is not yet fully understood, miR-21 prevented osteocyte apoptosis and showed a decreased expression in relation to age (37).

A cluster of three miRNAs, miR-23a, miR-24, and miR-27a promoted osteocyte differentiation. It did so by suppressing TGF-β signalling and reducing the expression of OCN. The net effect of this miRNA cluster was enhanced differentiation of terminal osteoblasts into osteocytes (168).

Lastly, osteocytes have shown to inhibit osteoblast maturation upon mechanical strain through targeting Insulin-like Growth Factor 1 (IGF-1) by miR-29b. Under normal circumstances, IGF-1 enhanced osteoblast differentiation (169). Interestingly, Zeng et al. showed that miR-29b is a mechanosensitive miRNA whose expression reduced upon mechanical strain. Mechanical strain reduced miR-29b expression and thereby increased IGF-1 secretion, resulting in enhanced osteoblastic differentiation and maturation in order to preserve or increase bone formation upon strain.

To date, not much research has been done into miRNAs related to osteocyte formation, functioning and survival. This warrants for more research into this cell type that is increasingly under attention in the field of bone regeneration.

### Chondrogenesis

Chondrogenesis plays an important role in fracture healing, as it is key to the formation of cartilage at the early stages of endochondral ossification. Chondrogenic differentiation and proliferation are two processes that are characteristic of different phases in fracture healing (1). Chondrogenesis has shown to be promoted by miR-29a, miR-140, miR-181a, miR-218, miR-222, miR-335, and miR-337. By directly targeting FOXO3A, miR-29a promoted the expression of P21 & P27. Increased expression of these proteins decreased cellular proliferation, but was required for MSC commitment toward chondrocytic lineage (170).

MiR-140, expressed specifically in cartilage tissue during embryonic development, targeted HDAC4. Under normal conditions, HDAC4 suppressed RUNX2, an important initiator of chondrocyte hypertrophy. Through this mechanism, miR-140 increased RUNX2 activity and thereby contributed to chondrocyte hypertrophy (171). *In vivo* work by Zheng et al. showed that miR-181a was expressed to a greater extent in the hypertrophic zone of human long bones during the developmental stage. Indeed, research showed that overexpression of miR-181a in human chondrocytes increased the expression of RUNX2, OSX, and OCN. By enhancing these transcription factors, miR-181a promoted chondrocyte hypertrophy (172). On the other hand, early chondrogenesis of MSCs was enhanced by miR-218 whilst suppressing chondrocytic maturation. MiR-218 directly targeted HPGD through which it regulated the expression of several chondrogenic, as well as osteogenic markers. Overexpression of miR-218 led to increased expression of SOX9, glycosaminoglycans and Cartilage Oligomeric Matrix Protein whilst reducing the expression of several osteogenic markers, such as ALP, COL1A1, OCN, and OPN (98).

Another interesting miRNA in the field of chondrogenesis is miR-335. It promoted chondrogenic differentiation via two independent mechanisms. On the one hand, it directly targeted DKK1 by which it enhanced β-catenin/TFC activity, on the other hand it directly targeted Dishevelled-associated Activator of Morphogenesis 1 & Rho associated Coiled-coil containing protein Kinase 1, resulting in enhanced SOX9 expression. Another interesting aspect about the mechanism of action behind miR-335 is that it promoted its own expression via a positive feedback loop (173). MiR-337 was also capable of enhancing chondrogenesis, presumably through targeting TGF-βR2, thereby promoting the differentiation and maturation of chondroblasts. MiR-337 overexpression resulted in increased expression of Aggrecan (AGC), whereas inhibition of miR-337 resulted in reduced AGC1 expression. Furthermore, miR-337 has shown to inhibit the activity of MMP3, an enzyme important in cartilage degeneration (174).

Contrary to the above-mentioned miRNAs, miR-1, miR-26b, miR-125b, miR-146a, miR-206, miR-214, and miR-222 have shown to inhibit chondrogenic differentiation. MiR-1 and miR-26b did so by inhibiting AGC synthesis. Sumiyoshi et al. showed that miR-1 primarily suppressed AGC expression in hypertrophic chondrocytes as compared to proliferating chondrocytes (175). MiR-26b also decreased AGC synthesis. This occurred through inhibition of the Wnt-signalling pathway (176). Under normal circumstances, miR-125b has shown to be downregulated in human BMSCs. Although no direct mechanism of action has been revealed yet, miR-125b attenuated IL-1B target gene expression whilst directly targeting OSX, thereby preventing proper chondrocyte differentiation (177). Another important signalling pathway in endochondral ossification is the TGF-β signalling pathway. MiR-146a affected TGF-β signalling by decreasing the expression of SMAD2 and SMAD3, which resulted in enhanced expression levels of RUNX2 and ALP and decreased SOX9 expression. MiR-146a thereby reduced chondrogenesis and steered toward osteogenic differentiation through enhanced RUNX2 and ALP activity (94).

MiR-206 inhibited chondrocyte proliferation, as seen by reduced expression levels of AGC and COL2A1, whilst enhancing apoptosis through decreasing Bcl2 expression. Simultaneously, the expression of RUNX2 and MMP13, also involved in angiogenic regulation by miR-191, increased upon miR-206 expression (178). Research by Roberto et al. revealed that certain miRNAs regulated chondrogenesis in a dose-dependent manner. An example is miR-214, vital to normal skeletal development and cartilage formation, although research revealed that miR-214 overexpression resulted in a substantial decrease in expression of SOX9, COL2A1 and Activating Transcription Factor 4 (179). Lastly, Yoshizuka et al. revealed that miR-222 interferes with angiogenesis, as described above, as well as chondrogenesis and osteoblastogenesis. Regarding chondrogenesis, miR-222 inhibition promoted chondrogenic differentiation in hMSCs, as confirmed by enhanced expression of COL2A1, AGC, and SOX9 (31).

## Discussion

This review displays the functions of miRNAs in fracture healing, providing a concise overview on regulatory roles of miRNAs in specific aspects that are related to bone regeneration. It therefore aids in determining possible future research aims, as well as offering a broad, integrated view on the role of miRNAs in cellular signalling related to fracture healing.

The literature describes the role of specific miRNAs in several processes that are involved in fracture healing. Proper fracture healing is dependent on the successful coherence of these processes. The complexity of cellular communication and functioning underline the dynamic and multifaceted nature of bone regeneration. MiRNAs play an important role in cellular communication and functioning, thereby greatly influencing fracture healing on several aspects. One single miRNA can affect the expression of several proteins, influencing multiple processes differentially within bone regeneration. Additionally, miRNA expression has shown to be dynamic, due to the gradual transitions in-between several phases of the fracture healing cascade, requiring differential cellular activities. Interestingly, various miRNAs influenced fracture healing processes in a contradictive manner, indicating the need for more research to clarify their role in bone regeneration. This may be related to spatiotemporal expressions.

Focusing on potential diagnostic or therapeutical applications, miRNAs have shown to be differentially expressed in several musculoskeletal diseases, indicating an involvement in bone formation and regeneration, as well as pathophysiological mechanisms. Furthermore, *in vivo* research, presented throughout this review, on the therapeutic application of miRNAs in bone regeneration has already shown promising results. However, more research on miRNAs, their targets, involvement in various pathways, and systemic responses is required to develop clinical applications.

Several challenges are evident regarding the clinical application of miRNAs. On a cellular level, examples of such challenges are the degradation by nucleases, endosomal entrapment, activation of innate immune responses, and low cellular membrane penetration (180). Furthermore, delivery of miRNAs to a target tissue, and delivery vectors are challenging. In fracture healing, one can think of local application during fracture surgery, but unwanted toxicity is a major component that needs to be taken into account. Looking at a systemic application of miRNAs, various barriers appear, such as the need for miRNAs to travel through the bloodstream, after which they have to extravasate and selectively enter a target tissue (181). Moreover, miRNAs can exhibit differential, tissue specific effects, since they are often involved in various pathways simultaneously, requiring in depth knowledge on cellular signalling on the individual patient level to minimise the risk of undesired side effects (182).

Although the role of miRNAs has been broadly researched in several specific aspects of bone regeneration, such as osteoblastogenesis, more insights should be gained in other aspects of fracture healing. One should be aware that the literature discussed throughout the present review concerns English literature only, has been selected on a limited amount of keywords, and was processed according to a set of strict exclusion criteria in order to maintain a focus on healthy fracture healing, therefore potentially missing out on specific literature on miRNAs related to skeletal pathophysiology. This indicates that fracture healing research would benefit from a more integrative approach, considering the sophisticated interplay of several processes as described above. This review therefore potentially serves as a benchmark, describing the intricate role of miRNAs throughout various processes within fracture healing, and offering useful insights for future research into the regulatory role of miRNAs in bone regeneration.

## Author Contributions

MG: idea. RG and JK: literature search and selection and reading literature. RG: writing manuscript. RG, JK, MG, MP, and TB: revising manuscript. All authors agree on their contribution and approved the submitted version.

## Conflict of Interest

The authors declare that the research was conducted in the absence of any commercial or financial relationships that could be construed as a potential conflict of interest.

## Publisher's Note

All claims expressed in this article are solely those of the authors and do not necessarily represent those of their affiliated organizations, or those of the publisher, the editors and the reviewers. Any product that may be evaluated in this article, or claim that may be made by its manufacturer, is not guaranteed or endorsed by the publisher.
